# Trends in Small Animal Reproduction: A Bibliometric Analysis of the Literature

**DOI:** 10.3390/ani12030336

**Published:** 2022-01-29

**Authors:** Penelope Banchi, Ada Rota, Alessia Bertero, Guillaume Domain, Hiba Ali Hassan, Joke Lannoo, Ann Van Soom

**Affiliations:** 1 Department of Internal Medicine, Reproduction and Population Medicine, Faculty of Veterinary Medicine, Ghent University, Salisburylaan 133, 9820 Merelbeke, Belgium; guillaume.domain@ugent.be (G.D.); hiba.alihassan@ugent.be (H.A.H.); joke.lannoo@ugent.be (J.L.); ann.vansoom@ugent.be (A.V.S.); 2 Department of Veterinary Sciences, University of Turin, Largo Paolo Braccini 2-5, 10095 Grugliasco, Italy; ada.rota@unito.it (A.R.); alessia.bertero@unito.it (A.B.)

**Keywords:** small animal, reproduction, canine, feline, bibliometric

## Abstract

**Simple Summary:**

Reproduction in small animals is an expanding research area, with focus on breeding improvement and clinical management of domestic carnivores. The aim of the present study was to conduct a bibliometric analysis of the literature of the last decade on small animal reproduction, to point out main sources, most prolific countries, and emerging and neglected topics. Results show that research in biotechnologies for assisted reproduction has a central and increasing role in this field. Diversity in author keywords was also pointed out and a consensus to better categorize research in this field is proposed to reduce this problem in the future.

**Abstract:**

Small animal reproduction (SAR) is a main research field in veterinary medicine and bibliometric analyses are useful to investigate trends in specific research areas. The objective of the present study was to conduct a bibliometric analysis of the literature of the last decade on SAR. A search equation was created, and documents were retrieved from the Web of Science database. Documents were manually revised, categorized and R software version 4.1.2 with Bibliometrix R package version 3.1 and MS Excel were used to perform the analyses. The included documents (*n* = 1470) were mainly research articles (78%). The top countries for the number of documents and citations were Brazil, United States, Italy, Poland, and Korea. These also account for the most prolific authors and institutions. Analyses by author keywords, categories, and recent reviews of the literature suggest that research on the canine species is more abundant than research on the feline one and that reproductive biotechnologies are a main research focus. Some clinical topics are still considered niche or neglected themes (e.g., semen collection in tomcats, neonatology). However, heterogeneity and ambiguity in keywords and categories are undeniable. This study offers interesting insights, providing definitions for main keywords in the field of SAR.

## 1. Introduction

In the field of veterinary sciences, small animal reproduction is becoming more important given the increased interest in breeding of companion animals [1] and in studying domestic carnivores as a model to develop biotechnologies for endangered species conservation [2]. Nowadays many organizations promote specialization in the field of veterinary medicine all around the world (e.g., the American College of Theriogenologists–ACT, and the European College of Animal Reproduction–ECAR). These organizations are recognizing different subspecialties, among which also small animal reproduction. Continuing education in this field is promoted by societies such as the European Veterinary Society for Small Animal Reproduction (EVSSAR). Conferences and meetings are regularly organized, and academics and practitioners are encouraged to share their knowledge and experience in this field, stimulating scientific curiosity and directing research to solve practical issues. Nevertheless, many black holes, unverified beliefs, and limitations still exist in this field [1] and despite the high number of published articles and reviews, some questions in the field of small animal reproduction remain unanswered. An interesting approach to investigate trends, hot topics, and neglected questions is represented by bibliometric analyses [3]. This kind of study allows for the analysis of a multitude of publications, pointing out dynamics regarding the impact of documents and journals, citation patterns, and weighting contributions to the literature and productivity of researchers and institutions within a specific area. This is useful to deduce future development of research and blind spots in a particular field. The publication of papers is estimated to increase by 4% every year [4]; hence, bibliometric analyses represent a perfect tool to keep pace with the new developments in veterinary medicine.

No previous bibliometric analysis of the literature in the field of small animal reproduction has ever been performed, according to the authors’ knowledge. Hence, the aim of the present study was to carry out a bibliometric analysis of the literature on small animal reproduction published in the last decade, to evaluate current trends and future perspectives in this continuously evolving area.

## 2. Materials and Methods

Publications were retrieved from the Web of Science (WoS) database on 4 January 2022 considering a time frame of 10 years (2012–2021).

The search equation was developed using a combination of key words belonging to the field of veterinary reproduction and Boolean operators as follow: (“REPRODUCTION” OR “REPRODUCTIVE” OR “FERTILITY” OR “UTERUS” OR “OVARIES” OR “OOCYTE” OR “TESTES” OR “SEMEN” OR “SPERM” OR “NEONATAL” OR “NEWBORN” OR “SPAYING” OR “MAMMARY” OR “PENIS” OR “VAGINA” OR “OVIDUCT” OR “PROSTATE” OR “PUPPIES” OR “KITTENS” OR “COLOSTRUM”) AND (“DOG” OR “BITCH” OR “CANINE” OR “CAT” OR “QUEEN” OR “FELINE”). WoS topic field were used to limit the search to the fields “TITLE”, “AUTHOR KEY WORDS”, and “ABSTRACT”. No filter regarding language was included, whereas results were filtered by publication type, including only research papers, reviews, meeting abstracts, proceeding papers, letters, and corrections. In order to minimize any mistakes or missing information and to identify the animal species analyzed in each document, a manual revision of the articles was performed by PB. Raw data were extracted in plain text format using the WoS extraction tool. Information fields related to authors, affiliations, journals, keywords, research areas, citations, titles, and abstracts were included in the extraction.

The bibliometric analysis of the Web of Science raw data was conducted using R software version 4.1.2 (R Foundation for Statistical Computing, Vienna, Austria) using Bibliometrix R package version 3.1 and its tool Biblioshiny [5]. This allowed us to estimate the contribution of journals, countries, and authors and to assess time trends, identifying the most cited papers and used words, in terms of author keywords and keywords plus. Keyword plus is a tool as effective as author keywords in bibliometric analyses, but it is less comprehensive in representing the article’s content [6]. Therefore, the analysis was mainly performed by author keywords. Country contribution was defined using the first author’s country. In addition, Bibliometrix provides mapping analysis to visualize relevant information such as keyword co-occurrences network maps. Graphs were created through Biblioshiny and Microsoft Excel (MS Excel vers. 16.49, Microsoft, Redmond, WA, USA). Documents were also manually sorted into categories (according to the “selected papers” section of the EVSSAR website). Research areas and types of documents were categorized using the WoS results analysis tool. The impact factors (IF) of the main journals were extracted from the latest Journal Citation Reports (JCR, 2020) by Clarivate Analytics. Finally, a list of the newest literature reviews regarding small animal reproduction, assessing hot topics in this field by analyzing keywords and subfields.

## 3. Results

### 3.1. General Information and Time Trends

**Figure 1:**
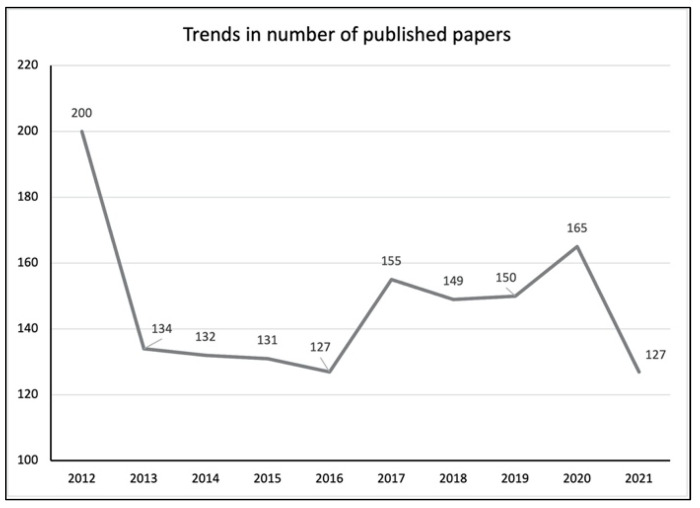
Trends of publication in the field of small animal reproduction. The trends are irregular, and the most prolific year was 2012 (*n* = 200), followed by 2020 (*n* = 165).

In total, 2856 documents matching the search criteria were retrieved. Only 1470 documents were deemed as pertinent to small animal reproduction. The annual evolution of publications during this time span is reported in Figure 1. The annual growth rate was4.92%; however, the trend was not regular, with 2012 being the most prolific year (*n* = 200) followed by 2020 (*n* = 165). The mean number of documents per year was 147 (±22.7 SD). Most of the retrieved publications were research articles (*n* = 1148, 78.1%), followed by meeting abstracts (*n* = 215, 14.6%), reviews (*n* = 48, 3.3%), proceeding papers (*n* = 36, 2.5%), corrections (*n* = 12, 0.8%), and letters (*n* = 11, 0.7%).

The distribution of the retrieved documents by research areas according to WoS is reported in Table 1 and majority of publications were included in the subject area “Veterinary Sciences” (*n* = 613, 41.7%). The canine species was more represented than the feline species (*n* = 937 and *n* = 479), although some papers focused on both (*n* = 54).

### 3.2. Sources

**Figure 2:**
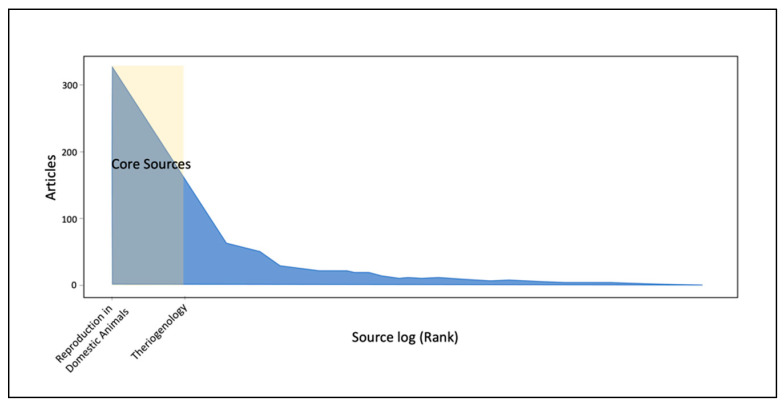
Bradford’s Law plot indicating the core sources of the literature in the field of small animal reproduction. Field-oriented journals (Reproduction in Domestic Animals and Theriogenology) account for most of the literature on small animal reproduction in the last decade.

The number of journals that published documents on small animal reproduction was 288. *Reproduction in Domestic Animals* (*n* = 334, 22.7%) and *Theriogenology* (*n* = 164, 11.2%) were the most prolific journals as well as the most cited, as confirmed by the Bradford’s Law plot, indicating these two journals as the core sources of the whole collection (Figure 2). Table 2 reports the twenty most prolific journals with their 2020 impact factor and rank. These sources published 34% of the papers regarding small animal reproduction between 2012 and 2021.

### 3.3. Authors and Affiliations Analysis

Authors involved in publications on small animal reproduction were 4874 in the last decade, with a mean of 3.32 authors per documents and 0.3 documents per author. Most of the documents were authored by more than one person (*n* = 1399, 95.2%), with only 71 single-authored documents (4.8%). Furthermore, 75% of the authors participated in just one publication (*n* = 3659), whereas 1040 authors appeared in two to five articles (21.4%) and only 175 authors (3.6%) participated in more than five publications. The co-authorship analysis, considering the ratio between the total number of authors of multi-authored papers and the total number of multi-authored papers, revealed a collaboration index of 3.44 and 5.53 co-authors per document.

Both academic and research institutes (*n* = 731) and private laboratories, veterinary clinics and hospitals (*n* = 325) contributed to the literature.

To analyze country contribution, the corresponding author’s affiliation was considered. A total number of 59 countries were involved in literature production with Brazil (*n* = 210, 14.2%, cit. = 1138), USA (*n* = 158, 10.7%, cit. = 1355), Italy (*n* = 127, 8.6%, cit. = 749), Poland (*n* = 90, 6.1%, cit. = 430), and Korea (*n* = 55, 3.7%, cit. = 332) being the top five countries for number of publications and citations. Furthermore, to estimate inter-country collaboration, the number of multiple country publications (MCP) was extracted and the MCP ratio (MPC articles/total publications per country) was calculated. The most prolific countries showed MCP ratios as follows: Korea (0.2364), Italy (0.2205), USA (0.1772), Brazil, and Poland (both 0.1667).

### 3.4. Most Relevant Papers and Citation Analysis

The impact of publications in the field of small animal reproduction was assessed analyzing data from citation indexes. A total of 30,635 references were included in the 1470 retrieved documents, whit a mean number of 5.59 citations per document and a total of 5769 articles citing the ones included in the present bibliometric analysis. Papers that received more than 10 citations were 269, representing 18.3% of the total. Most of the documents received 1 to 10 citations (*n* = 740, 50.3%), whereas 31.4% (*n* = 461) of the documents did not receive any citation.

The twenty most cited papers are reported in Table 3, they account for 15% of the citations for this collection (*n* = 864) and they were published by 15 journals.

The journals *Theriogenology* and *Reproduction in Domestic Animals* contributed, respectively, with six and four papers to the list of the most relevant documents (Table 3), accounting for 24.9% (*n* = 1440) and 20.6% (*n* = 1187) of the citations, respectively, for a total number of citations equal to 2627 (45.5%).

### 3.5. Analysis by Keywords

**Figure 3:**
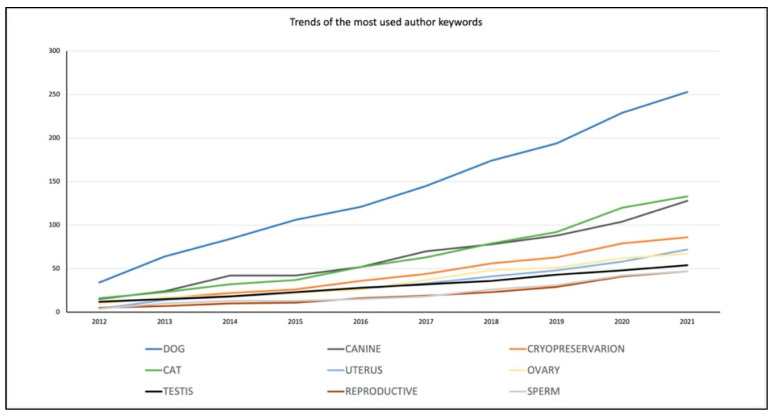
Trends of the most-used author keywords in the field of small animal reproduction in the decade 2012–2021.

The total number of keywords chosen by authors for this collection was 2536, with dog (*n* = 327), canine (*n* = 131), cryopreservation (*n* = 86), uterus (*n* = 72), ovary (*n* = 67), feline (*n* = 61), testis (*n* = 54), reproduction (*n* = 47), sperm (*n* = 47), and semen (*n* = 42) being the most reported authors keywords. The trends of the most common author keywords in the decade 2012–2021 are shown in Figure 3. Keyword plus were also retrieved (*n* = 3268) as they are a WoS tool for capturing the content and scientific concepts presented in articles. In this case, spermatozoa (*n* = 120), dogs (*n* = 114), cryopreservation (*n* = 108), motility (*n* = 71), semen (*n* = 57), cat (*n* = 54), fertility (*n* = 51), artificial insemination (*n* = 50), oxidative stress (*n* = 50), and fertilization (*n* = 47) were the most frequent.

When these keywords are grouped according to Medical Subject Headings (MeSH controlled vocabulary) the frequencies of appearance in the author’s keywords changes slightly, as reported in Table 4.

**Figure 4:**
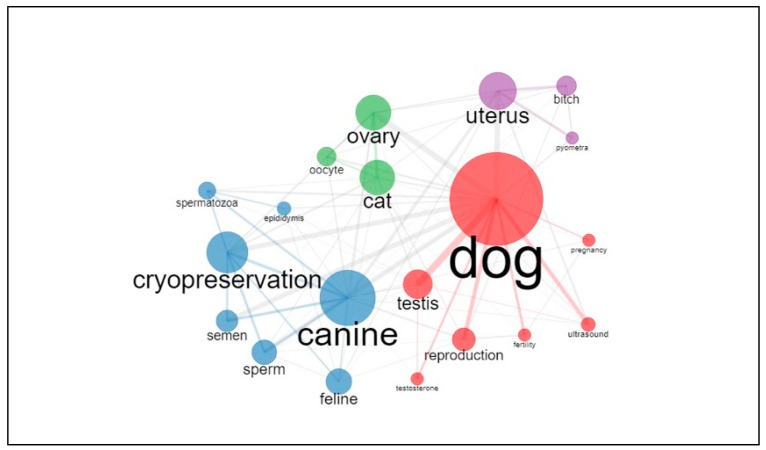
Co-occurrence analysis of the most frequent author keywords in the field of small animal reproduction. Keywords are grouped in four clusters and the thickness of the lines connecting keywords represents the strength of their relationship. The diameter of nodes is proportional to the frequency of each keyword.

Figure 4 shows the co-occurrence analysis of the most frequent author keywords. The size of the node is proportional to the frequency of the keyword, while the thickness of the line connecting two keywords represents the strength of the relationship. The four colors represent the different clusters revealed by the network analysis.

**Figure 5:**
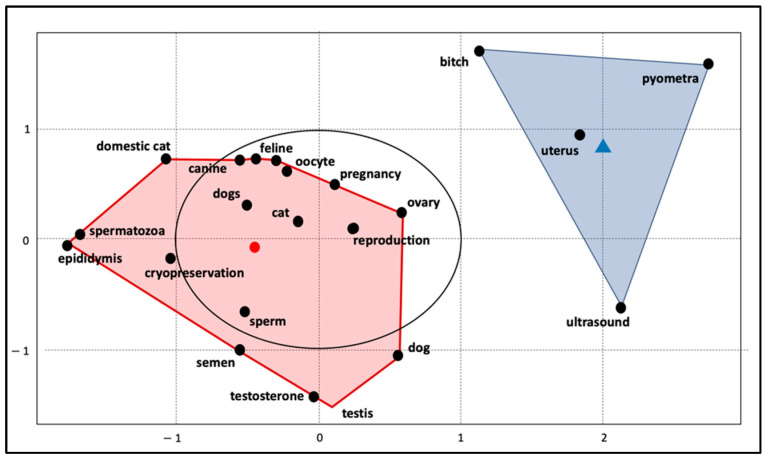
Clustering map (multifactorial analysis) of author keywords in the field of small animal reproduction. The main words are grouped in two clusters. The center of the map (black circle) represents the center of the research field.

A multifactorial analysis was also performed to see how the most frequent author keywords relate. Therefore, Figure 5 shows that the most used keywords in the field of small animal reproduction are grouped in just two main clusters. Nodes are close to each other when a large proportion of documents treat them together.

**Figure 6:**
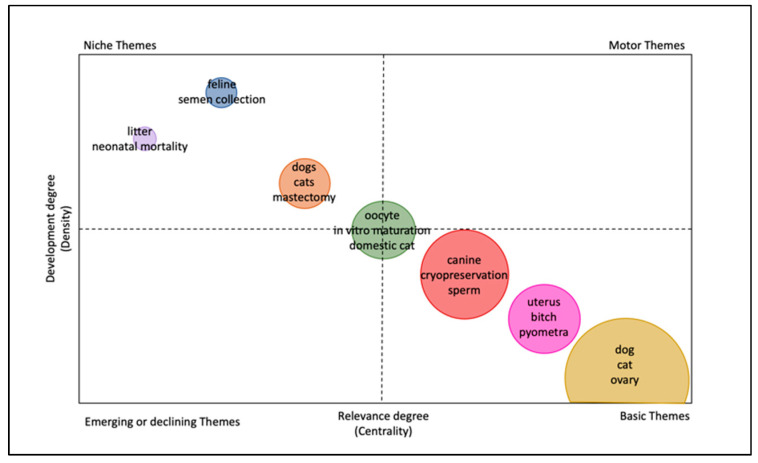
Thematic map showing relevance and development of topics in small animal reproduction.

A thematic map was created by using a two-dimensional matrix considering centrality and density as measurements to highlight the most important topics in the field of small animal reproduction. Centrality measures the relevance of a topic, while density estimates the development of a theme. The upper right quadrant is occupied by important and well-developed themes, lower left emerging or declining themes, upper left well-developed themes, and lower right transversal basic themes. The clusters are represented by bubbles within the map, which are labeled by keywords with the highest occurrences, and their size is proportional to the keyword occurrence (Figure 6).

**Figure 7:**
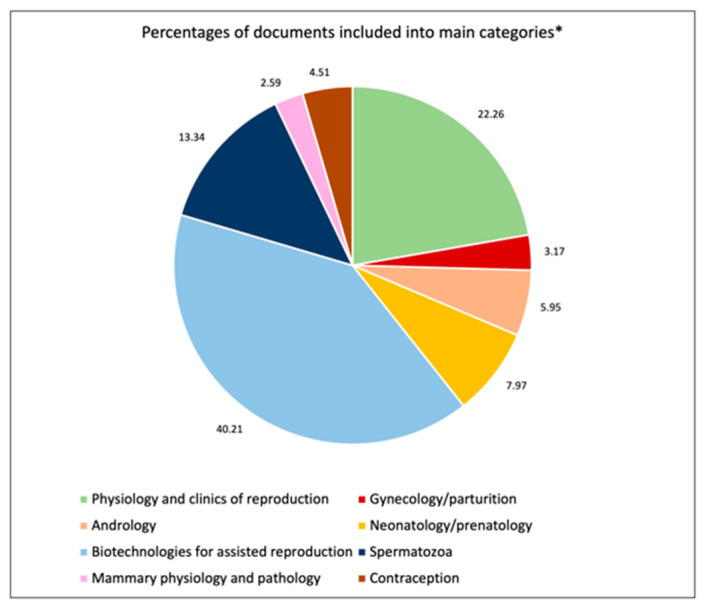
Distribution of documents included in the present study into EVSSAR (European Veterinary Society for Small Animal Reproduction) “selected papers” section (2020). *: “Biotechnologies for assisted reproduction” and “Physiology and clinics of reproduction” are the categories that include the highest percentage of documents.

Finally, the sorting of documents into EVSSAR categories (Figure 7) show that “Biotechnologies for assisted reproduction in carnivores” and “Physiology and clinics of reproduction” are the main fields of recent research in small animal reproduction (40.2% and 22.2%, respectively).

### 3.6. Latest Reviews in the Field of Small Animal Reproduction

Literature reviews are the keystone of evidence-based medicine, and they are essential to select further research topics, to have an idea on the state-of-the-art in specific areas. The collection analyzed in the present study included 49 reviews. The review articles published in the last 5 years on small animal reproduction (title, author, year, journal, subfield, author keywords) are reported in Table 5, to emphasize topics on which scientists recently decided to focus to address the need of summarizing scientific knowledge. Three main subfields were identified: biotechnologies, clinical sciences, and physiology. Eleven papers were assigned to the subfield “clinical sciences” (47.9%), nine papers to the subfield “biotechnologies” (39.1%), and three documents were included in the subfield “physiology” (13%).

## 4. Discussion

The present bibliometric analysis had the objective of exploring contributions, trends, impact, and dynamics in research on small animal reproduction. During the last decade, research in this field has decreased based on this analysis. At first sight, this may seem counter intuitive, but it must be noted that 2020 has so far been the second most prolific year ever in small animal reproduction research. If we evaluate the mean number of publications per year of the previous decade (2002–2011), it is obvious that this number is lower than in the last decade (118.6 ± 39.5 and 147 ± 22.7, respectively), suggesting a generally increasing interest in research on small animal reproduction.

Main research areas according to WoS were ‘Veterinary Sciences’ and ‘Reproductive Biology’. Although, journals in WoS can be assigned to multiple research areas and this can lead to ambiguous classification of papers into research areas. This could explain why many papers concerning canine semen were assigned to the research area ‘Agricultural Dairy Animal Science’.

Most of the documents were research articles and the theme-oriented journals *Reproduction in Domestic Animals* and *Theriogenology* were the core sources of the collection. Brazil, USA, Italy, Poland, and Korea were the most productive countries, also showing the highest inter-country collaboration rate. These countries account for almost 50% of the total scientific research about small animal reproduction. Accordingly, the universities of Milan (Italy), Wroclaw (Poland), and São Paulo (Brazil) were the most prolific, also being the affiliations of the most cited authors in this collection.

Literature on the canine species doubles that in the feline species, although this is not surprising as it reflects the numbers of published papers on dogs and cats in general (154,535 in dogs versus 67,568 in cats, according to research on WoS). Furthermore, the trend lines show that research on canine reproduction is also increasing more than research on feline reproduction in terms of the number of published papers. However, the importance of the feline species as a topic of research in animal reproduction is increasing, especially in relation to the interest in studying cats to improve technologies for assisted reproduction (ARTs) in endangered wild felids. Most of the wild felids are considered at risk, and cats represent a good model to develop research in this area [50]. This is emphasized by the fact that the keywords “cat”, “oocyte”, “ovary”, “vitrification”, and “in vitro maturation” belong to the same cluster in our analysis, meaning that these keywords often appear together in recently published papers. Furthermore, they represent a very central theme in research. The interest in dogs as a research model for wild canids conservation is limited for two reasons: there are not many endangered species [50] and assisted reproductive biotechnologies such as in vitro fertilization (IVF) are at present no routine practice in dogs [2]. Nevertheless, the keywords “dog” and “canine” are clustered with terms belonging to the spheres of gynecology (uterus, pyometra), andrology (testis, testosterone, spermatogenesis), with many appearances also in the area of male biotechnologies (cryopreservation, sperm, epididymis, CASA—Computer Assisted Sperm Analysis, sperm quality, semen). This is not surprising given the high interest in the use and conservation of dog semen. The interest lies especially in maximizing the profit of breeders and, most of all, of in improving the availability of genetic material to guarantee diversity [51]. The recurrence of the keywords indicating the feline species is lower in this area. However, research on biotechnologies in tomcats is very actual and was targeted by multiple reviews of the literature, underlining the interest of researchers for the topic [28,43,44], even if it still considered as a niche subject. In general, reproductive biotechnologies represent an important area of research in small animal reproduction, surpassing research on clinical aspects of reproduction by a factor of two. This could be associated with the practical and ethical limitations in conducting clinical research compared to the fast collection and processing of samples in the field of biotechnologies [52], along with the already cited interests in applying assisted reproductive technologies to other species and increasing breeding performances. The present study also shows how some themes are considered the core of small animal reproduction, with topics such as uterine pathologies, pyometra in the bitch, and canine semen being the focus of extensive research in the last decade. On the other hand, literature on neonatology (i.e., studies regarding puppies and kittens from birth to weaning) in general seems to be lacking and topics such as neonatal mortality are considered niche themes. However, neonatology and pediatrics represent everyday interests for veterinarians, breeders, and pet owners and neonatal mortality is still considered a main issue in domestic carnivores [53,54].

A huge heterogeneity in keywords used by the authors was noticed, with the most reported word (“dog”), being chosen only 314 times, and double if we also considered the MeSH terms. However, not all databases are using controlled vocabulary and therefore, working on a consensus on keywords use should be encouraged. This is the reason why we chose to perform a further analysis of the included documents by sorting them into EVSSAR “selected papers” categories. The sorting was manually performed, but many documents could be sorted into more than one category. For example, many articles about semen could be classified as “Biotechnology for assisted reproduction, Spermatozoa”, and even “Andrology”. Additionally, articles about artificial insemination could be categorized both as “Physiology and clinics of reproduction” and “Biotechnologies for assisted reproduction”.

Hereafter we propose keywords based on EVSSAR “selected papers” categories. These keywords should be used in research on small animal reproduction to classify papers into main topics. Definitions are also provided.

Physiology of reproduction: Research on the function of healthy reproductive organs, tissues, cells, molecules, and chemical processes concerning small animal reproduction.

Gynecology: Any clinical practice dealing with the female reproductive system of small animals, including diseases of the female reproductive organs and mammary glands, endocrinological changes of the normal cycle, breeding soundness evaluation and infertility, determination of the phase of the cycle, and any pathology of pregnancy, parturition and post-partum period.

Andrology: Any clinical practice dealing with the male reproductive system of small animals, including diseases of the male reproductive organs, endocrinological deviations, breeding soundness evaluation and infertility, semen analysis with no intervention or modification on the semen by the operator after collection.

Biotechnology for assisted reproduction: Technologies applied to germ cells, gametes, and embryos with an effect of small animal reproduction. This includes practice and research on techniques including artificial insemination, embryo transfer, in vitro maturation, in vitro fertilization, in vitro production, intracytoplasmic sperm-injection, cryopreservation of sperm, oocytes and embryos, sperm and embryo sexing, cloning, nucleus transfer, and gene transfer.

Contraception: Artificial (medical or surgical) techniques aimed to prevent pregnancy in small animals.

Neonatology: Treatment and care of newborn kittens and puppies from birth to weaning.

The most cited documents of this bibliometric analysis belong to the field of small animal reproduction, but some of them represent crossroads with fields such as Infectious Diseases, Behavior, Anesthesiology, and Pathology. In fact, the paper with the highest number of citations is “*Coxiella burnetiid associated reproductive disorders in domestic animals–a critical review*” by [7]. This paper focuses on domestic animals in general and the canine and feline species only have a marginal role in it. Nevertheless, *Coxiella burnetii* is an intracellular bacterium causing Q fever in humans and reproductive disorders in mammals. Periparturient cats have been implicated in several outbreaks of Q fever in humans; therefore, the importance of this topic is undeniable, and the focus on public health justify the high number of citations. However, all the other relevant papers focus on the canine and feline species, deepening the fields of Clinical Sciences, Physiology, and Biotechnology.

It needs to be mentioned that the results for the research equations included a great number of papers on canine and feline mammary tumors, representing more than 40% of the whole collection. Papers were checked by the authors and only the ones deemed as pertinent to small animal reproduction were included in the analysis. Specifically, papers focusing on the association between mammary tumors and the presence of sexual steroids, on the effect of spaying on mammary neoplasms, on the co-occurrence with other reproductive pathologies, and papers clinically and surgically oriented were included, whereas articles strictly related to pathology, histology, immunohistochemistry, and chemotherapy were excluded. Research on mammary tumors in small animals is extensive, not only for the high incidence of mammary neoplasms in domestic carnivores [55,56], but also for the role of the canine and feline species as a model for human breast cancer [57,58]. This explains the high degree of development of this research area, and the decision to select only papers fitting the field of small animal reproduction was mandatory in order to avoid a huge bias in results. Additionally, the revision of the included papers helped in avoiding the biases deriving from the sole use of tools such as Bibliometrix and WoS. A possible limitation of the present study is that the research was conducted solely in WoS [59] in a limited time frame (2012–2021), leading to the exclusion of papers and sources not included in WoS or published before 2012, with possible underestimation of the comprehensive literature.

## 5. Conclusions

The present study is the first bibliometric analysis in the field of small animal reproduction, offering interesting insights about research evolution and development, pointing out what topics are considered basic knowledge in this area, with consolidated data and extensive research, what research areas are currently neglected, and what are the emerging topics that scientists are currently investigating, such as canine and feline biotechnologies. Furthermore, we provide definitions for keywords to be used for papers and database searches in the field of small animal reproduction with the aim of adding homogeneity to this complex and wide field of research.

## Figures and Tables

**Table 1:** Distribution of the retrieved documents by research areas according to Web of Science.

Veterinary Sciences	613
Reproductive Biology	433
Agriculture Dairy Animal Science	190
Developmental Biology	73
Zoology	64
Genetics Heredity	30
Cell Biology	30
Pathology	21
Biology	9
Physiology	7

**Table 2:** The top twenty of most prolific journals publishing papers in the field of small animal reproduction.

Journal	IF ^a^	Rank and Category Quartile ^b^
*Reproduction in Domestic Animals*	2.005	Veterinary Sciences: 48/146 (Q2); Reproductive Biology: 27/30 (Q4); Agriculture, Dairy & Animal Science: 27/63 (Q2)
*Theriogenology*	2.740	Veterinary Sciences: 21/146 (Q1); Reproductive Biology: 20/30 (Q3)
*Animal Reproduction Science*	2.145	Veterinary Sciences: 43/146 (Q2); Reproductive Biology: 26/30 (Q4); Agriculture, Dairy & Animal Science: 23/63 (Q2)
*Animals*	2.752	Veterinary Sciences: 19/146 (Q1); Agriculture, Dairy & Animal Science: 13/63 (Q1)
*Arquivo Brasileiro de Medicina Veterinaria Zootecnia*	0.442	Veterinary Sciences: 129/146 (Q4)
*Journal of Feline Medicine and Surgery*	2.015	Veterinary Sciences: 47/146 (Q2)
*Reproduction Fertility and Development*	2.311	Reproductive Biology: 24/30 (Q4); Zoology: 39/174 (Q1); Developmental Biology: 27/41 (Q3)
*Journal of Veterinary Medical Science*	1.267	Veterinary Sciences: 88/146 (Q3)
*Polish Journal of Veterinary Sciences*	0.821	Veterinary Sciences: 106/146 (Q3)
*Acta Scientiae Veterinariae*	0.332	Veterinary Sciences: 136/146 (Q4)
*Cryobiology*	0.68	Biology: 44/93 (Q2); Physiology: 50/81 (Q3)
*BMC Veterinary Research*	2.741	Veterinary Sciences: 20/146 (Q1)
*Veterinary Journal*	2.688	Veterinary Sciences: 26/146 (Q1)
*Frontiers in Veterinary Science*	3.412	Veterinary Sciences: 9/146 (Q1)
*Pesquisa Veterinaria Brasileira*	0.584	Veterinary Sciences: 118/146 (Q4)
*PLoS ONE*	3.24	Multidisciplinary Sciences: 26/72 (Q2)
*Veterinary Clinics of North America–Small Animal Practice*	2.093	Veterinary Sciences: 45/146 (Q2)
*Acta Veterinaria Scandinavica*	1.695	Veterinary Sciences: 58/146 (Q2)
*Journal of Reproduction and Development*	2.214	Agriculture, Dairy & Animal Science: 22/63 (Q2); Reproductive Biology: 25/30 (Q4)
*Medycyna Weterynaryjna-Veterinary Medicine-Science and Practice*	0.383	Veterinary Sciences: 131/146 (Q4)

IF ^a^: 2020 impact factor based on Journal Citation Report (includes self-citations). ^b^: by impact factor.

**Table 3:** The top twenty most-cited articles in the field of small animal reproduction.

Title	Author	Year	Journal	IF ^a^	TC
Coxiella burnetiid associated reproductive disorders in domestic animals–a critical review [7]	Agerholm, J.S.	2013	*Acta Veterinaria Scandinavica*	1.695	137
Variation in reproductive traits of members of the genus Canis with special attention to the domestic dog (*Canis familiaris*) [8]	Lord, K. et al.	2013	*Behavioural Processes*	1.777	83
Reproductive capability is associated with lifespan and cause of death in companion dogs [9]	Hoffman, J.M. et al.	2013	*PLoS ONE*	3.24	67
Canine perinatal mortality: A cohort study of 224 breeds [10]	Tønnessen, R. et al.	2012	*Theriogenology*	2.74	59
In vivo survival of domestic cat oocytes after vitrification, intracytoplasmatic sperm injection and embryo transfer [11]	Pope, C.E. et al.	2012	*Theriogenology*	2.74	43
Epidermal growth factor (EGF) sustains in vitro primordial follicle viability by enhancing stromal pathways in the prepubertal, but not adult cat ovary [12]	Fujihara, M. et al.	2014	*Biology of Reproduction*	4.285	40
Evaluation of testicular artery blood flow by Doppler ultrasonography as a predictor of spermatogenesis in the dog [13]	Zelli, R. et al.	2013	*Research in Veterinary Science*	2.534	38
Effects of tramadol alone, in combination with meloxicam or dipyrone, on postoperative pain and the analgesic requirement in dogs undergoing unilateral mastectomy with or without ovariohysterectomy [14]	Teixeira, R.C.R. et al.	2013	*Veterinary Anaesthesia and Analgesia*	1.648	37
Expression of genes involved in the embryo-maternal interaction in the early-pregnant canine uterus [15]	Kautz, E. et al.	2014	*Reproduction*	3.906	35
Chemical composition of lipids present in cat and dog oocyte by Matrix-Assisted Desorption Ionization Mass Spectometry (MALDI-MS) [16]	Apparicio, M. et al.	2012	*Reproduction in Domestic Animals*	2.005	33
Concomitant canine distemper, infectious canine hepatitis, canine parvoviral enteritis, canine infectious tracheobronchitis, and toxoplasmosis in a puppy [17]	Headley, S.A. et al.	2013	*Journal of Veterinary Diagnostic Investigation*	1.279	33
Oxidative stress at different stages of two-step semen cryopreservation procedures in dogs [18]	Lucio, C.F. et al.	2016	*Theriogenology*	2.74	31
Vaccination against Feline Panleukopenia: implications from a field study in kittens [19]	Jakel, V. et al.	2012	*BMC Veterinary Research*	2.741	31
Poor owner knowledge of feline reproduction contributes to the high proportion of accidental litters born to UK pet cats [20]	Welsh, C.P. et al.	2014	*Veterinary Record*	2.695	30
Colour and pulsed doppler ultrasonographic study of the canine testis [21]	Carrillo, J.D. et al.	2012	*Reproduction in Domestic Animals*	2.005	30
Computer-Assisted Sperm Analysis in dogs and cats: an update after 20 years [22]	Rijsselaere, T. et al.	2012	*Reproduction in Domestic Animals*	2.005	29
Fecal microbiota transplantation in puppies with canine parvovirus infection [23]	Pereira, G.Q., et al.	2018	*Journal of Veterinary Internal Medicine*	3.333	29
Gene expression profiling of pluripotency and differentiation-related markers in cat oocytes and preimplantation embryos [24]	Filliers, M. et al.	2012	*Reproduction, Fertility and Development*	2.311	27
Evaluation of the prevalence of urinary incontinence in spayed female dogs: 566 cases (2003-2008) [25]	Forsee, K.M. et al.	2013	*Journal of the American Veterinary Medical Association*	1.936	26
Effects of the GnRH analogue deslorelin implants on reproduction in female domestic cats [26]	Toydemir, T.S.F. et al.	2012	*Theriogenology*	2.74	26

IF ^a^: Impact Factor from the 2020 Journal Citation Reports; TC: total citations.

**Table 4:** Authors’ keywords (AKW) grouped according to controlled vocabulary (MeSH) and their frequencies. Frequencies of Keyword plus (KP) are also reported.

Words	Frequency AKW	KP
Canine, canines, dog, dogs	615	363
Sperm, spermatozoa, sperms, spermatozoon, spermatozoas, spermatozoons, semen	328	465
Cats, cat, felines, feline	313	266
Reproduction, reproductions, reproductive, reproductively, reproductives, reproductivity	151	42
Ovary, ovaries, ovarian	109	79
Oocyte, oocytes, oocytic	102	133
Cryopreservation, cryopreservability, cryopreservable, cryopreservated, cryopreserved, cryopreservations, cryopreservative, cryopreservatives, cryopreserve, cryopreserving	95	142
Uterus, uteri	99	81
Teste, testi, testis, testes	78	102
Bitch, bitches	65	80

**Table 5:** Review articles published in the last 5 years in the field of small animal reproduction.

Title	Author	Year	Journal	Subfield ^a^ (and AUK)
Assisted reproductive techniques for canines: preservation of genetic material in domestic dogs [27]	Suzuki, H. et al.	2021	*Journal of Reproduction and Development*	Biotechnologies (assisted reproductive technique; cryopreservation, dog, embryo, sexing, spermatozoa)
Canine and feline epididymal semen–A plentiful source of gametes [28]	Ali Hassan, H. et al.	2021	*Animals*	Biotechnologies (epididymis; spermatozoa; epididymal semen; maturation; collection)
Fighting like cats and dogs: challenges in domestic carnivore oocyte development and promises of innovative culture systems [29]	Colombo, M. et al.	2021	*Animals*	Biotechnologies (canine; feline; co-culture; IVC; IVF; IVM; microfluidic; 3D)
Overview on the antioxidants, egg yolk alternatives, and mesenchymal stem cells and derivates used in canine sperm cryopreservation [30]	Mahiddine, F.Y. and Kim, M.J.	2021	*Animals*	Biotechnologies (dog; semen; cryopreservation; assisted reproductive technology)
Ovarian cysts in the bitch: an update [31]	Sasidharan, J.K. et al.	2021	*Topics in Companion Animal Medicine*	Clinical Sciences (ovary, follicular cyst; luteal cyst; canine; cystic endometrial hyperplasia-pyometra)
Canine brucellosis: an update [32]	Santos, R.L. et al.	2021	*Frontiers in Veterinary Sciences*	Clinical Sciences (Brucella canis; brucellosis; dog; abortion; reproductive diseases; zoonoses)
Antioxidants in assisted reproductive technologies: An overview on dog, cat, and horse [33]	Ciani, F. et al.	2021	*Journal of Advanced Veterinary and Animal Research*	Biotechnologies (antioxidants; assisted reproductive technologies (ARTs); animal reproduction; oxidative stress (OS)
The potential of aquaporins and connexins in dogs and their relation to the reproductive tract [34]	Kulus, M. et al.	2021	*Medycina Weterynaryjna–Science and Practice*	Physiology (aquaporins; connexins; reproduction; canine)
Normal and abnormal response to sperm deposition in female dogs: A review and new hypotheses for endometritis [35]	England, G.C.W. et al.	2021	*Theriogenology*	Clinical Sciences (dog; sperm; transport; reservoir; endometritis)
Lactation-related mammary gland pathologies–A neglected emergency in the bitch [36]	Vasiu, I. et al.	2021	*Reproduction in Domestic Animals*	Clinical Sciences (animal breeding; dogs; cats; general reproduction; gynaecology; obstetrics; pathology; species)
Uterine serosal inclusion cysts in the dog–case report and literature review of canine uterine cysts [37]	Sievert, M. et al.	2020	*Tieraerztliche Praxis Ausgabe Kleintiere Heimtiere*	Clinical Sciences (cyst; uteropathy; bitch; reproduction)
Exosomes as a potential tool for supporting canine oocyte development [38]	Lee, S.H. and Saadeldin, I.M.	2020	*Animals*	Physiology (oviduct; dog; exosomes; extracellular vesicles; oocytes development)
Monitoring ovarian function and detecting pregnancy in felids: A review [39]	Andrews, C.J. et al.	2020	*Theriogenology*	Clinical Sciences (cat; estrus; fecal metabolite; ovulation; reproductive state)
Canine spermatozoa–What do we know about their morphology and physiology? An overview [40]	Chlopik A, C.J. and Wysokinska, A.	2020	*Reproduction in Domestic Animals*	Biotechnologies (dog; epididymis; sperm morphology; sperm physiology; spermatozoa)
Development of dog immune system: from in uterus to elderly [41]	Pereira, M. et al.	2019	*Veterinary Sciences*	Physiology (dog; immune system; immunity; passive immune transfer; immunity development)
Canine ovarian gonadoblastoma with dysgerminoma overgrowth: a case study and literature review [42]	Flores, A.R. et al.	2019	*Journal of Ovarian Research*	Clinical Sciences (bitch; ovary; gonadoblastoma; dysgerminoma; sex cord-stromal tumour; immunohistochemistry; karyotype)
Influence of cooling temperature in sperm subpopulations of domestic cats [43]	Souza, A.K. et al.	2018	*Animal Reproduction Science*	Biotechnologies (cryopreservation; feline; spermatic subpopulation; multivariate statistics; spermatic kinetics)
Protocols for sperm cryopreservation in the domestic cat: A review [44]	Baranaamnuay, K.	2017	*Animal Reproduction Science*	Biotechnologies (feline; semen; freexing; method)
Effect of cabergoline on thyroid hormones and semen quality of dogs [45]	Mogheiseh, A. et al.	2017	*Topics in Companion Animal Medicine*	Biotechnologies (canine; prolactin; semen; thyroxine)
Reproductive medicine and neonatology of dog and cat [46]	Georgiev, P.	2017	*Tieraerztliche Praxis Ausgabe Kleintiere Heimtiere*	Clinical Sciences (not available); article in German
Effect of melatonin on the reproductive cycle in female cats: a review of clinical experiences and previous studies [47]	Schafer-Somi, S.	2017	*Journal of Feline Medicine and Surgery*	Clinical Sciences (not available)
Cystic ovaries and ovarian neoplasia in the female dog—A systematic review [48]	Arlt, SP. and Heimerl, P.	2016	*Reproduction in Domestic Animals*	Clinical Sciences (not available)
Pyometra in the queen. To spay or not to spay? [49]	Hollinshead, F. and Krekeler, N.	2016	*Journal of Feline Medicine and Surgery*	Clinical Sciences (not available)

AUK: Author Keywords. ^a^ Clinical sciences, biotechnologies, Physiology.

## Data Availability

Publicly available datasets were analyzed in this study. This data can be found here: https://www.webofscience.com (accessed on 14 November 2021).
